# Awake Prone Positioning for Non-Intubated COVID-19 Patients with Acute Respiratory Failure: A Meta-Analysis of Randomised Controlled Trials

**DOI:** 10.3390/jcm12030926

**Published:** 2023-01-25

**Authors:** Huzaifa Ahmad Cheema, Amna Siddiqui, Sidhant Ochani, Alishba Adnan, Mahnoor Sukaina, Ramsha Haider, Abia Shahid, Mohammad Ebad Ur Rehman, Rehmat Ullah Awan, Harpreet Singh, Natalie Duric, Brigitta Fazzini, Antoni Torres, Tamas Szakmany

**Affiliations:** 1 Intensive Care Unit, Department of Chest Medicine, King Edward Medical University, Lahore 54000, Pakistan; 2 Department of Medicine, Karachi Medical and Dental College, Karachi 74700, Pakistan; 3 Department of Medicine, Khairpur Medical College, Khairpur 66020, Pakistan; 4 Department of Medicine, Rawalpindi Medical University, Rawalpindi 46000, Pakistan; 5 Department of Medicine, Ochsner Rush Medical Center, Meridian, MS 39301, USA; 6 Division of Pulmonary and Critical Care, Medical College of Wisconsin, Milwaukee, WI 53226, USA; 7 Critical Care Directorate, The Grange University Hospital, Aneurin Bevan University Health Board, Cwmbran NP44 2XJ, UK; 8 Adult Critical Care Unit, The Royal London Hospital, Barts Health NHS Trust, London E1 1BB, UK; 9 Department of Pneumology, Respiratory Institute, Hospital Clinic of Barcelona, 08036 Barcelona, Spain; 10 CibeRes (Centro de Investigación Biomédica en Red de Enfermedades Respiratorias, 06/06/0028), Institut d’Investigacions Biomèdiques August Pi i Sunyer (IDIBAPS), 28029 Barcelona, Spain; 11 School of Medicine, University of Barcelona, 08036 Barcelona, Spain; 12 Department of Anaesthesia, Intensive Care and Pain Medicine, Division of Population Medicine, Cardiff University, Cardiff CF14 4XN, UK

**Keywords:** awake prone positioning, COVID-19, acute respiratory failure, intubation, mortality

## Abstract

Introduction: Awake prone positioning (APP) has been widely applied in non-intubated patients with COVID-19-related acute hypoxemic respiratory failure. However, the results from randomised controlled trials (RCTs) are inconsistent. We performed a meta-analysis to assess the efficacy and safety of APP and to identify the subpopulations that may benefit the most from it. Methods: We searched five electronic databases from inception to August 2022 (PROSPERO registration: CRD42022342426). We included only RCTs comparing APP with supine positioning or standard of care with no prone positioning. Our primary outcomes were the risk of intubation and all-cause mortality. Secondary outcomes included the need for escalating respiratory support, length of ICU and hospital stay, ventilation-free days, and adverse events. Results: We included 11 RCTs and showed that APP reduced the risk of requiring intubation in the overall population (RR 0.84, 95% CI: 0.74–0.95; moderate certainty). Following the subgroup analyses, a greater benefit was observed in two patient cohorts: those receiving a higher level of respiratory support (compared with those receiving conventional oxygen therapy) and those in intensive care unit (ICU) settings (compared to patients in non-ICU settings). APP did not decrease the risk of mortality (RR 0.93, 95% CI: 0.77–1.11; moderate certainty) and did not increase the risk of adverse events. Conclusions: In patients with COVID-19-related acute hypoxemic respiratory failure, APP likely reduced the risk of requiring intubation, but failed to demonstrate a reduction in overall mortality risk. The benefits of APP are most noticeable in those requiring a higher level of respiratory support in an ICU environment.

## 1. Introduction

Patients with acute respiratory failure secondary to moderate to severe Coronavirus Disease 2019 (COVID-19) often require non-invasive respiratory support [1]. Unfortunately, the failure rate of non-invasive methods requiring escalation to invasive respiratory support is estimated to be between 34% and 44% [2,3]. Consequently, techniques such as awake prone positioning (APP) have been proposed as a supportive adjunct therapy to non-invasive respiratory support as a means of reducing the need for escalation to mechanical ventilation [4]. Despite significant improvements in patient outcomes since the beginning of the pandemic, invasive respiratory support still carries a significant risk of morbidity and mortality [5].

Prone positioning is a well-established treatment modality in patients with moderate and severe acute respiratory distress syndrome (ARDS) undergoing mechanical ventilation. It has been shown to significantly reduce mortality through a purported reduction in ventilation/perfusion mismatching, hypoxemia, and shunting [6,7].

With this in mind, studies have been conducted to observe the effect of APP in patients with respiratory failure secondary to COVID-19 infection. It was found that APP is feasible and well-tolerated in most patients who are awake and spontaneously breathing [8]. The findings regarding improved oxygenation are positive; however, there are a scarcity of data relating to important clinical outcomes such as intubation rates and death [9,10].

Previous systematic reviews have been limited by the inclusion of mostly underpowered randomized controlled trials (RCTs) conducted in the earlier phase of the pandemic [11,12], or the inclusion of observational studies that are prone to confounding bias [12,13]. Recently, the largest RCT to date, the COVI-PRONE (Awake Prone Position in Hypoxemic Patients with Coronavirus Disease 19) trial was published, which reported a non-significant reduction in intubation rates in the prone positioning group [14]. Owing to the availability of newer data and the limitations of prior meta-analyses, we conducted this meta-analysis to evaluate the efficacy and safety of APP in COVID-19 patients with acute hypoxemic respiratory failure.

## 2. Methods

This meta-analysis was conducted in accordance with the Preferred Reporting Items for Systematic Reviews and Meta-Analysis (PRISMA) guidelines (Supplementary Table S1) [15]. This study did not require ethical approval. Our protocol was registered with PROSPERO, The International Prospective Register of Systematic Reviews (CRD42022342426).

### 2.1. Data Sources and Search Strategy

An electronic search was performed on the Cochrane Central Register of Controlled Trials (CENTRAL via the Cochrane Library), MEDLINE (via PubMed), Embase (via Ovid), clinicaltrials.gov, and ProQuest Dissertations and Theses Global (PQDT) from inception up to August 2022. MeSH terms and relevant keywords for (prone position*) AND (awake or non-intubated) AND (COVID-19 OR SARS-CoV-2) were used. The detailed search strategy is available in Supplementary Table S2. Hand-searching of pertinent review articles and bibliographies of the included original articles were also undertaken. We performed forward citation searching using the Web of Science to identify further eligible articles.

### 2.2. Study Selection and Eligibility Criteria

All of the articles were imported into Mendeley Desktop 1.19.8 (Mendeley Ltd., Amsterdam, Netherlands) and duplicates were removed. Two authors (A.S. and A.A.) independently screened the titles and abstracts of all of the retrieved articles and removed those not fulfilling the inclusion criteria. Full texts of the remaining articles were reviewed against the eligibility criteria. Conflicts or disagreements were discussed and resolved with a third author (H.A.C.). We included RCTs comparing APP with supine positioning or standard of care with no prone positioning for non-intubated adult (>18 years old) patients with COVID-19-related acute hypoxemic respiratory failure. We did not apply any language restriction. We excluded articles that evaluated patients intubated before or at enrolment, and those including paediatric patients (<18 years of age). We also excluded case reports, observational studies, reviews and editorials, and articles that did not report any of our pre-specified outcomes.

### 2.3. Outcomes

The co-primary outcomes of our meta-analysis were the risk of intubation and the reported all-cause mortality in patients with COVID-19-related acute respiratory failure, while secondary outcomes included (1) the need for escalating respiratory support, (2) length of ICU stay, (3) length of hospital stay, (4) ventilation free-days, and (5) adverse events.

### 2.4. Data Extraction

For baseline characteristics, data extraction was done by two independent groups of authors, which included author names, year of publication, country, RCTs enrolment centres or location, patient enrolment, details including outcome measures for intervention and control groups, age, gender, BMI, corticosteroids use, and follow-up days. Data were then checked by a third independent group of authors. For categorical outcomes, the number of events for that outcome and the total number of patients were extracted, while for continuous outcomes, the sample size, mean, or median were extracted as provided in the studies and the medians were converted to means for data analysis [16].

### 2.5. Risk of Bias and Certainty of Evidence Assessment

Two independent authors (S.O. and R.H.) assessed the risk of bias in the included studies using the Cochrane “Risk of bias” tool for randomized trials (RoB 2.0) [17]. RoB 2.0 addressed five specific domains: (1) bias arising from the randomization process, (2) bias due to deviations from intended intervention, (3) bias due to missing outcome data, (4) bias in measurement of the outcome, and (5) bias in the selection of the reported results. We applied this tool to each included study and the source of bias was graded as high, low, or unclear, which determined the risk of bias as high, low, or some concerns. Disagreements were discussed and resolved with a third author (M.S.). The quality of evidence was graded as very low, low, moderate, or high using the Grades of Recommendation, Assessment, Development, and Evaluation (GRADE) assessment tool on the basis of risk of bias, publication bias, imprecision, inconsistency, and indirectness [18].

### 2.6. Data Synthesis

Risk ratios (RR) and mean differences (MDs) for dichotomous and continuous outcomes, respectively, with 95% confidence intervals (CIs), were pooled using the DerSimonian and Laird random-effects model. The pooled results were represented graphically as forest plots. The Chi^2^ test and the I^2^ statistic were used to assess heterogeneity across studies. I^2^ values were interpreted according to the *Cochrane Handbook for Systematic Reviews of Interventions*, section 10.10 [19]. *p* < 0.10 was considered statistically significant for the Chi^2^ test [19]. We conducted the statistical analysis using Review Manager (RevMan, Version 5.4; The Cochrane Collaboration, Copenhagen, Denmark). We used Funnel plots and Egger’s test to assess publication bias when at least 10 studies were included in a meta-analysis, using the Jamovi (version 1.8; Jamovi, Sydney, Australia) MAJOR module, which is based on the metafor package for R [20].

We conducted subgroup analyses on our primary outcomes based on the respiratory support level and the patient location at enrolment. With regards to the respiratory support level, conventional oxygen therapy was defined as oxygen therapy without positive pressure such as a nasal cannula or a mask, and a higher level of respiratory support was defined as the use of positive airway pressure via high-flow nasal cannula or non-invasive ventilation. The location at enrolment was ICU versus non-ICU. Intermediate ICU or the emergency department was classified as ICU, whereas non-ICU indicated general hospital wards. *p*-value < 0.10 was considered statistically significant for the test for subgroup differences [21]. We also performed a post hoc exploratory meta-regression analysis, using the OpenMetaAnalyst software, under the random-effects model for our primary outcomes with the duration of APP in the intervention group as the covariate.

## 3. Results

### 3.1. Search Results

**Figure 1:**
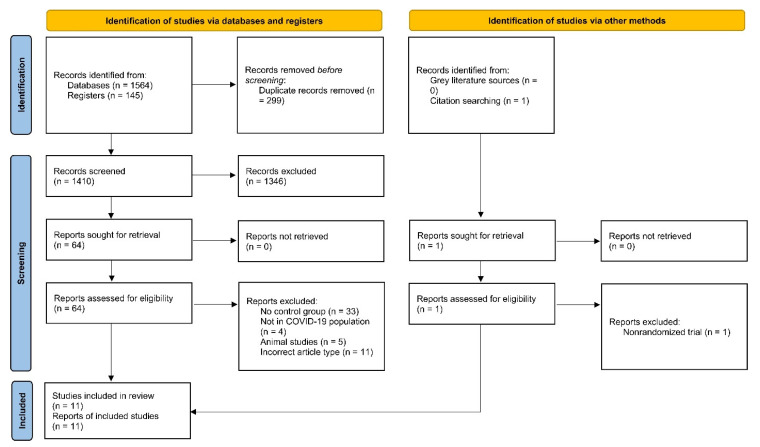
PRISMA 2020 flowchart.

The literature search retrieved 1712 studies. After the exclusion of duplicates, reviews, and ineligible articles, we included a total of 11 RCTs with a cumulative sample size of 2385 patients (1218 in the APP group and 1167 in the control group) in our review [14,22,23,24,25,26,27,28,29,30,31]. The literature screening process is summarised in Figure 1.

### 3.2. Characteristics of Included Studies

All of the studies were published between 2020 and 2022. There were five multicentre studies, four single-centre studies, and one multinational RCT. The APP procedures were of a variable duration in the included studies, ranging from 1 h to 16 h, or up to the tolerance of the patient. The follow-up duration ranged from 28 to 30 days for most trials; one trial had a follow-up of 1 day only [24], one of 14 days [27] and only one RCT had a follow-up of greater than 30 days (60 days) [14]. All of the included studies used different types of initial respiratory support. In five studies, patients were given a lower level of respiratory support (i.e., conventional oxygen therapy) [14,24,28,29,31]. Five studies used a higher level of respiratory support (i.e., high-flow nasal cannula or NIV) [22,23,26,27,30]. Patients were exclusively in an ICU setting in one study [26], exclusively in a general ward setting in six RCTs [23,24,25,27,29,31], and in both settings in one RCT [22]. In the study by Ehrmann et al., patients were in a mixed setting of ICU, intermediate care unit, emergency department, and general ward [30]. In the study by Gad et al., patients were in the critical care isolation unit [28]. In the study by Alhazzani et al., a monitored acute care unit was used [14]. The detailed characteristics of included studies are shown in Table 1.

### 3.3. Quality Assessment of Included Studies

Assessment of risk of bias using RoB 2.0 found a high risk of bias in three studies and some concerns of bias in six studies (Supplementary Figure S1). The most common issue was in the domains of the randomization process and deviations from intended interventions. The remaining two RCTs were judged to be at a low risk of bias [14,30].

### 3.4. Results of Meta-Analysis

#### 3.4.1. Primary Outcomes

##### Risk of Intubation

**Figure 2:**
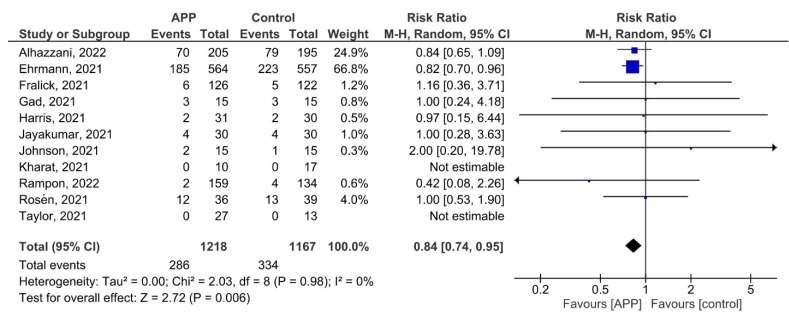
Effect of APP on the risk of intubation [14,22,24,25,26,27,28,29,30,31].

All 11 studies reported the need for intubation as an outcome. Patients in the APP group were at a significantly lower risk of needing intubation compared with the control group (RR 0.84, 95% CI: 0.74–0.95; *p* = 0.98, I^2^ = 0%; Figure 2). Egger’s test for funnel plot asymmetry did not demonstrate any suspicion of publication bias (*p* = 0.553; Supplementary Figure S2). The quality of evidence was judged to be moderate, with concerns about the risk of bias in the included studies (Supplementary Table S3).

In the subgroup analysis for the type of respiratory support at enrolment, a significant reduction in the need for intubation was reported in the APP group versus the control group for a higher level of respiratory support (RR 0.82, 95% CI 0.71–0.93), but not for conventional oxygen therapy (RR 1.07, 95% CI 0.66–1.73). However, there was no significant difference between the two subgroups (*P_interaction_* = 0.29; Supplementary Figure S3).

In the subgroup analysis for enrolment location, a significant reduction in the need for intubation was reported in the APP group versus the control group for the ICU setting (RR 0.83, 95% CI 0.73–0.95), but not for the non-ICU setting (RR 0.88, 95% CI 0.44–1.76). However, the test for subgroup differences was not significant (*P_interaction_* = 0.87; Supplementary Figure S4).

The results of meta-regression showed a non-significant negative association of APP duration with the risk of intubation, with longer durations of APP demonstrating a possible trend towards a greater benefit (coefficient = −0.033; *p* = 0.160; Supplementary Figure S5).

##### Mortality

**Figure 3:**
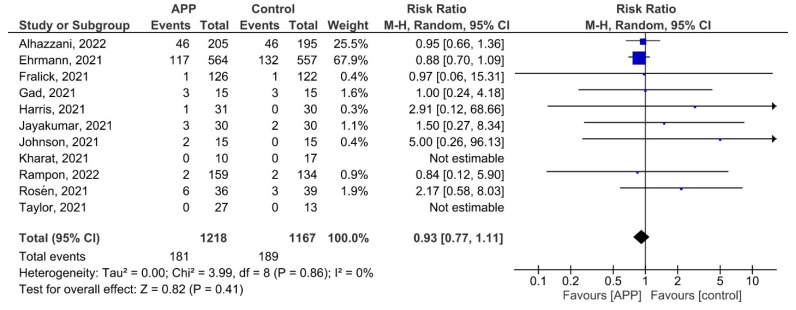
Effect of APP on the risk of all-cause mortality [14,22,24,25,26,27,28,29,30,31].

All 11 studies reported the incidence of all-cause mortality. Risk for all-cause mortality was comparable for the APP versus the control group (RR 0.93, 95% CI 0.77–1.11; *p* = 0.87; I^2^ = 0%; Figure 3). Egger’s test did not show any evidence of publication bias (*p* = 0.204; Supplementary Figure S6). The quality of evidence was judged to be moderate with concerns of imprecision and risk of bias (Supplementary Table S3).

In the subgroup analysis for the type of respiratory support, we found no difference for APP versus the control group in patients on a higher level of respiratory support (RR 0.92, 95% CI 0.76–1.10) as well as patients on conventional oxygen therapy (RR 1.14, 95% CI 0.47–2.75; *P_interaction_* = 0.64; Supplementary Figure S7).

In the subgroup analysis for enrolment location, there was no difference for APP versus the control group in patients admitted to the ICU (RR 0.91, 95% CI 0.75–1.10) and patients not admitted to the ICU (RR 0.81, 95% CI 0.41–1.59; *P_interaction_* = 0.75; Supplementary Figure S8).

In the meta-regression, there was no significant association between the duration of APP and the risk of all-cause mortality (coefficient = 0.006; *p* = 0.870; Supplementary Figure S9).

#### 3.4.2. Secondary Outcomes

##### Length of ICU Stay

The length of ICU stay was reported by five studies [22,23,26,28,30]. No significant difference was observed for APP versus the control group (MD 0.08, 95% CI 0.96–1.12; *p* = 0.88, I^2^ = 8%; Supplementary Figure S10). The quality of evidence was judged to be moderate with concerns of imprecision only (Supplementary Table S3).

##### Length of Hospital Stay

The length of hospital stay was reported by eight studies [22,23,24,25,27,28,30,31]. There was no significant difference between the two groups (MD 0.55, 95% CI −0.55–1.66; *p* = 0.33, I^2^ = 34%; Supplementary Figure S11). The quality of evidence was judged to be moderate with concerns of risk of bias (Supplementary Table S3).

##### Need for Escalating Respiratory Support

Nine studies reported the need for escalating respiratory support as an outcome [22,23,24,25,26,27,28,29,30]. No significant difference was detected between APP and the control group (RR 0.97, 95% CI 0.79–1.20; *p* = 0.77, I^2^ = 29%; Supplementary Figure S12). The quality of evidence was downgraded to moderate with concerns of risk of bias (Supplementary Table S3).

##### Ventilator-Free Days

Three studies reported ventilator-free days as an outcome [14,22,25]. Our analysis reported no significant difference between APP and the control group (MD 3.36, 95% CI 7.20–13.92; *p* = 0.53, I^2^ = 95%; Supplementary Figure S13). The quality of evidence was downgraded to very low with concerns of inconsistency, imprecision, and risk of bias (Supplementary Table S3).

##### Adverse Events

For safety outcomes, the APP group and the control group were comparable in terms of risk of adverse events and serious adverse events (RR 1.29, 95% CI 0.52–3.21; *p* = 0.59, I^2^ = 76% and RR 1.60, 95% CI 0.94–2.73; *p* = 0.08, I^2^ = 0%, respectively; Supplementary Figures S14 and S15). No publication bias was detected in the outcome of the incidence of adverse events (*p* for Egger’s = 0.173; Supplementary Figure S16). The quality of evidence for the risk of adverse events was downgraded to very low with concerns of imprecision, inconsistency, and risk of bias, while for serious adverse events it was judged to be moderate with concerns of imprecision only (Supplementary Table S3).

## 4. Discussion

The results of our meta-analysis demonstrate a likely reduction in the risk of intubation with APP with no increase in the incidence of total adverse or serious adverse events. In other important patient-centred outcomes, APP and control groups were comparable in terms of all cause-mortality, ICU- length of stay, ventilator-free days, and the need for escalating respiratory support. The setting where APP was delivered appeared to influence the effectiveness. Patients admitted to the ICU and those receiving a higher level of respiratory support, including high-flow nasal oxygen, showed a reduction in risk of intubation with APP, while patients receiving conventional respiratory support and those not admitted to the ICU showed no benefit with APP.

Prone positioning improves oxygen delivery and may reduce mortality in patients on mechanical ventilation suffering from severe ARDS [7,32,33]. The specific pathophysiological effects of APP in viral pneumonia are not yet fully understood. The observed improvements in oxygenation suggest that similar mechanisms are at play in patients who are mechanically ventilated. In prone positioning, the gravitational shift in the thoracic cavity enhances the reopening of poorly ventilated atelectatic areas and promotes the recruitment of dorsal-dependent lung regions [34]. APP in spontaneously breathing patients may promote more homogeneous diffusion and dispersal of pleural pressure, reducing the strain on the lungs in acute hypoxaemia [8,30,35,36]. The improvement in ventilation/perfusion (V/Q) matching can also be attributed to the redistribution of blood flow due to gravitational forces to the better-ventilated areas and during the APP session due to the relaxation of hypoxic pulmonary vasoconstriction, leading to a better right ventricular performance [37,38,39]. Additionally, APP is associated with a decrease in the fluid collection in the alveoli, which may improve the hypoxemic state and supply of adequate oxygen throughout the lungs [30,40,41]. These beneficial changes in V/Q matching could help with improvement in oxygenation, ameliorating the high respiratory drive and reducing the risk of self-inflicted lung injury, leading to reduced risk of intubation in COVID-19 patients.

Our results, which are significantly influenced by the meta-trial by Ehrmann et al. [30], are consistent with a significant reduction in the risk of intubation, similar to a previous meta-analysis by Li et al. [11]. This reduced risk of intubation, however, failed to translate into a reduction in mortality with APP. Notably, our results do not agree with the previously published meta-analyses that report a reduction in mortality in the APP group [12,13,42]. We only included RCTs in our meta-analysis, thus removing the confounding and selection biases that these previous meta-analyses suffered from.

Our results and Li et al. both highlight a trend towards a greater benefit of APP for patients in the ICU and patients receiving a higher level of respiratory support. Several factors such as an increased staff-to-patient ratio and close respiratory monitoring promoting greater adherence may account for the different efficacy of APP in ICU versus non-ICU settings, and higher level versus conventional respiratory support. Nevertheless, these findings should be interpreted with caution as the difference between the subgroups was not significant in our study. This may be attributed to low power due to the limited sample size in each subgroup.

Interestingly, the RCT by Alhazzani et al. [14], which had the second highest weightage in our meta-analysis, showed that prone positioning may not be advantageous for patients with a more severe disease, which is in contrast with the results of the meta-trial by Ehrmann et al. [30]. A recent study highlighted that response to prone positioning is significantly different when atelectatic areas change to dense consolidation [43]. As these changes develop over a variable time period, the observed difference in the trials may be attributed to the different inclusion criteria and APP protocols.

The duration of prone positioning varied considerably in the included studies, ranging from 1–2 h/day to 8–10 h/day; however, most studies had an APP duration of less than 8 h/day. In our meta-regression analysis, increased duration of APP was associated with a lower risk of intubation; however, as this trend did not achieve statistical significance, it should be interpreted with due caution. Nevertheless, a recent study showed that an increased APP duration to 8 h/day was associated with better clinical outcomes [44]. Future RCTs should attempt to investigate this association further to determine the optimal duration of APP.

There is a concern about complications such as device displacement, pressure ulcers, and hemodynamic instability during APP [45]. In our meta-analysis, we found that the rate of adverse events was similar between the two groups, albeit with substantial imprecision, which does not rule out an increased risk of serious complications.

There are several barriers to APP in complex hospital settings, as noted by multiple authors of the studies included in this meta-analysis. Patients’ hesitation, discomfort, lack of knowledge, and pregnancy itself can be potential barriers and the current literature shows poor adherence towards APP [46]. It has been shown that musculoskeletal pain and other discomforts are common in severe COVID-19 disease, and this increases the risk of non-adherence even with relatively simple medical procedures [47]. Another possible barrier can be a lack of knowledge, perception, and attitude towards APP, as has been the case with prone positioning of mechanically ventilated patients [45]. Additionally, APP can be a labour-intensive procedure and requires careful respiratory monitoring to recognise therapy failure and avoid harm from self-inflicted lung injury and late intubation. Moreover, leadership and team dynamics play an essential role, and thus team discomfort and lack of experience with APP along with prior negative experiences and observed adverse effects of the intervention could be significant barriers to APP [48].

### Limitations

Our study has several limitations that need to be considered while interpreting our results. The findings of our meta-analysis might not be generalizable to non-COVID-related acute hypoxemic respiratory failure as it includes patients with COVID-19 only. Furthermore, some patients in the control group followed prone positioning for a few hours, while some patients in the prone positioning group reverted to the supine position to attain a comfortable position [25,26,27]. Additionally, values quoted by studies for actual awake prone positioning were unmethodical as it was observed and recorded inconsistently with unknown accuracy by bedside clinicians. Patient adherence to the APP protocol must be objectively measured in any future studies conducted. To achieve this, the start and end times for each position, including prone, lateral, and supine positions, must be accurately recorded.

## 5. Conclusions

Our meta-analysis showed that APP likely lowers the risk of intubation in COVID-19 patients with acute hypoxemic respiratory failure, with no increase in the incidence of adverse events. The risk of mortality, length of stay, and length of mechanical ventilation is not affected by APP; however, the studies are underpowered and heterogeneous for these outcomes. Future large-scale RCTs are required to confirm these findings and identify the subpopulations, degree of disease severity, and optimal duration in order to benefit the most from APP. 

## Figures and Tables

**Table 1:** Characteristics of the included studies.

Study	Country	Setting	Participants		Age *		Gender, *n* (%)		BMI *		Baseline P/F (S/F) *		Baseline Corticosteroid Use, *n* (%)		Duration of APP in the Intervention Group (hours) *	Follow-up
			Control	APP	Control	APP	Control	APP	Control	APP	Control	APP	Control	APP		
Alhazzani, W., 2022 [14]	Canada, Kuwait, Saudi Arabia, and USA	(ICU) or a monitored acute care unit.	400		58.3 ± 13.2	56.8 ± 12.5	61 (31) Female	56 (27) Female	29.5 ± 4.9	29.7 ± 4.7	136 (110–181)	132 (103–174)	186 (95)	194 (95)	Daily: 5 (2.6–8)	At 30 and 60 days
			195	205			134 (69) Male	149 (73) Male								
Ehrmann, S., 2021 [30]	Mexico, France, USA, Spain, Ireland, and Canada	ICU, intermediate care unit, emergency department, general ward	1126		60.7 ± 14	61.5 ± 13.3	191 (34) Female	184 (33) Female	29.7 ± 4.6	29.7 ± 4.6	148.6 ± 43.1	147.9 ± 43.9	492 (88%)	494 (88%)	Daily: 5 (1.6–8.8)	28 days
			557	564			366 (66) Male	380 (67) Male								
Fralick, M., 2021 [29]	USA and Canada	General ward	257		54 (44–62)	59.5 (45–68)	45 (36.9) Female	44 (34.9) Female	NR		305 (267, 339)	303 (261, 336)	119 (97.5)	117 (92.9)	Total: 6 (1.5–12.8)	Up until mortality, hospital discharge or 30 days
															Daily: 2.5	
			122	126												
Gad, G.S., 2021 [28]	Egypt	Critical care isolation	30		46 (33–51)	49 (38–62)	Male:Female		NR		11 (97–175)	126 (88–164)	NR		NR	NR
			15	15			08:07	09:06								
Rampon, G., 2022 [27]	USA	General ward	134	159	54 (43–63)	52 (39–62)	80 (59.7) Male	96 (60.4) Male	NR		402 (311–457)	396 (308–457)	NR		NR	14 days
Harris, unpublished	Qatar	General ward	30	31	40 (36–45)	41 (35–50)	25 (83.3) Male	29 (93.5) Male	27.2 ± 4.6	28.4 ± 3.7	196 (182–240)	196 (165–245	30 (100)	31 (100)	NR	30 days
Jayakumar, D., 2021 [26]	India	ICU	30	30	57.3 ± 12.1	54.8 ± 11.1	25 (83.3) Male	25 (83.3) Male	25.8 ± 2.6	28.2 ± 5.7	P/F 185.6 ± 126.1	P/F 201.4 ± 118.8	30 (100)	30 (100)	NR	Until Discharge
Johnson, S.A., 2021 [25]	USA	General ward	15	15	46 (33–51)	49 (38–62)	8 (53.3) Male	9 (60) Male	29.3 (24.4–32.9)	32.9 (27.5–39.4)	NR		NR		Total: 1.6 (0.2–3.1)	28 days
Kharat, A., 2021 [24]	Switzerland	General ward	27		58 ± 12		17 (63) Total		28.2 ± 4.7		336 (303–388)	318 (284–341)	NR		Total: 4.9 ± 3.6	1 day (24 h)
			17	10			11 (65) Male	6 (60) Male	27.3 ± 4.2	29.7 ± 5.3						
Rosén, J., 2021 [22]	Sweden	ICU and ward	75		65 (55–70)	66 (53–74)	32 (82) Male	23 (64) Male	29 (27–33)	28 (25–30)	115.5 (93.75–129.75)	115.5 (86.25–130.5)	NR		Daily: 9 (4.4–10.6)	30 days
Taylor, S.P., 2021 [31]	USA	General ward	41		60 (54–63)	56 (45–66)	3 (23) Female	10 (37) Female	31 (28–38)	29 (26–39)	NR		NR		NR	Until discharge

* Data are reported as mean ± SD or median (IQR). APP, awake prone positioning; BMI, body mass index; ICU, intensive care unit; P/F, ratio of partial pressure of arterial oxygen to fraction of inhaled oxygen; S/F, ratio of pulse oxygen saturation to fraction of inhaled oxygen; NR, not reported.

## Data Availability

The data that support the findings of this study are available from the corresponding author upon reasonable request.

## Supplementary Materials

The following supporting information can be downloaded at: https://www.mdpi.com/article/10.3390/jcm12030926/s1, Figure S1: Risk of bias assessment of included studies; Figure S2: Funnel plot for risk of intubation; Figure S3: Subgroup analysis of risk of intubation for type of respiratory support before randomization; Figure S4: Subgroup analysis of risk of intubation for enrolment location; Figure S5: Meta-regression for risk of intubation by APP duration; Figure S6: Funnel plot for all-cause mortality; Figure S7: Subgroup analysis of all-cause mortality for type of respiratory support before randomization; Figure S8: Subgroup analysis of all-cause mortality for enrolment location; Figure S9: Meta-regression for risk of all-cause mortality by APP duration; Figure S10: Effect of APP on length of ICU stay; Figure S11: Effect of APP on length of hospital stay; Figure S12: Effect of APP on need for escalating respiratory support; Figure S13: Effect of APP on ventilator-free days; Figure S14: Effect of APP on the incidence of adverse events; Figure S15: Effect of APP on the incidence of serious adverse events; Figure S16: Funnel plot for the incidence of adverse events; Table S1: PRISMA 2020 checklist; Table S2: Search strategy for PubMed which was adapted for other databases; Table S3: Grading of recommendations assessment, development, and evaluation (GRADE) summary of findings.
