# Supplementary material for: Awake Prone Positioning for Non-Intubated COVID-19 Patients with Acute Respiratory Failure: A Meta-Analysis of Randomised Controlled Trials
Source: J Clin Med. 2023 Jan 25;12(3):926. doi: 10.3390/jcm12030926 (PMC9917863; doi:10.3390/jcm12030926)

# Supplementary Material

**Awake Prone Positioning for Non-Intubated COVID-19 Patients with Acute Respiratory Failure: A Meta-Analysis of Randomised Controlled Trials**

This supplemental material has been provided by the authors to give readers additional information about their work.

**Supplementary Table S1. PRISMA 2020 checklist**

| Section and Topic | Item # | Checklist item | Reported on page # |
|---|---|---|---|
| **TITLE** | | | |
| Title | 1 | Identify the report as a systematic review. | 1 |
| **ABSTRACT** | | | |
| Abstract | 2 | See the PRISMA 2020 for Abstracts checklist. | 3 |
| **INTRODUCTION** | | | |
| Rationale | 3 | Describe the rationale for the review in the context of existing knowledge. | 4,5 |
| Objectives | 4 | Provide an explicit statement of the objective(s) or question(s) the review addresses. | 5 |
| **METHODS** | | | |
| Eligibility criteria | 5 | Specify the inclusion and exclusion criteria for the review and how studies were grouped for the syntheses. | 6 |
| Information sources | 6 | Specify all databases, registers, websites, organisations, reference lists and other sources searched or consulted to identify studies. Specify the date when each source was last searched or consulted. | 5 |
| Search strategy | 7 | Present the full search strategies for all databases, registers and websites, including any filters and limits used. | Suppl. |
| Selection process | 8 | Specify the methods used to decide whether a study met the inclusion criteria of the review, including how many reviewers screened each record and each report retrieved, whether they worked independently, and if applicable, details of automation tools used in the process. | 6 |
| Data collection process | 9 | Specify the methods used to collect data from reports, including how many reviewers collected data from each report, whether they worked independently, any processes for obtaining or confirming data from study investigators, and if applicable, details of automation tools used in the process. | 6 |
| Data items | 10a | List and define all outcomes for which data were sought. Specify whether all results that were compatible with each outcome domain in each study were sought (e.g. for all measures, time points, analyses), and if not, the methods used to decide which results to collect. | 6 |
| | 10b | List and define all other variables for which data were sought (e.g. participant and intervention characteristics, funding sources). Describe any assumptions made about any missing or unclear information. | 6 |
| Study risk of bias assessment | 11 | Specify the methods used to assess risk of bias in the included studies, including details of the tool(s) used, how many reviewers assessed each study and whether they worked independently, and if applicable, details of automation tools used in the process. | 7 |
| Effect measures | 12 | Specify for each outcome the effect measure(s) (e.g. risk ratio, mean difference) used in the synthesis or presentation of results. | 7 |

| Section and Topic | Item # | Checklist item | Reported on page # |
|---|---|---|---|
| Synthesis methods | 13a | Describe the processes used to decide which studies were eligible for each synthesis (e.g. tabulating the study intervention characteristics and comparing against the planned groups for each synthesis (item #5)). | 7 |
| | 13b | Describe any methods required to prepare the data for presentation or synthesis, such as handling of missing summary statistics, or data conversions. | 7 |
| | 13c | Describe any methods used to tabulate or visually display results of individual studies and syntheses. | 7 |
| | 13d | Describe any methods used to synthesize results and provide a rationale for the choice(s). If meta-analysis was performed, describe the model(s), method(s) to identify the presence and extent of statistical heterogeneity, and software package(s) used. | 7 |
| | 13e | Describe any methods used to explore possible causes of heterogeneity among study results (e.g. subgroup analysis, meta-regression). | 8 |
| | 13f | Describe any sensitivity analyses conducted to assess robustness of the synthesized results. | NA |
| Reporting bias assessment | 14 | Describe any methods used to assess risk of bias due to missing results in a synthesis (arising from reporting biases). | 7 |
| Certainty assessment | 15 | Describe any methods used to assess certainty (or confidence) in the body of evidence for an outcome. | 7 |
| **RESULTS** | | | |
| Study selection | 16a | Describe the results of the search and selection process, from the number of records identified in the search to the number of studies included in the review, ideally using a flow diagram. | 8, Figure 1 |
| | 16b | Cite studies that might appear to meet the inclusion criteria, but which were excluded, and explain why they were excluded. | Figure 1 |
| Study characteristics | 17 | Cite each included study and present its characteristics. | 8,9 suppl. |
| Risk of bias in studies | 18 | Present assessments of risk of bias for each included study. | 9, Table 1 |
| Results of individual studies | 19 | For all outcomes, present, for each study: (a) summary statistics for each group (where appropriate) and (b) an effect estimate and its precision (e.g. confidence/credible interval), ideally using structured tables or plots. | Figures, suppl. |
| Results of syntheses | 20a | For each synthesis, briefly summarise the characteristics and risk of bias among contributing studies. | 10-12 |
| | 20b | Present results of all statistical syntheses conducted. If meta-analysis was done, present for each the summary estimate and its precision (e.g. confidence/credible interval) and measures of statistical heterogeneity. If comparing groups, describe the direction of the effect. | 10-12 |

| Section and Topic | Item # | Checklist item | Reported on page # |
|---|---|---|---|
| | 20c | Present results of all investigations of possible causes of heterogeneity among study results. | 10,11 |
| | 20d | Present results of all sensitivity analyses conducted to assess the robustness of the synthesized results. | NA |
| Reporting biases | 21 | Present assessments of risk of bias due to missing results (arising from reporting biases) for each synthesis assessed. | 10,12 |
| Certainty of evidence | 22 | Present assessments of certainty (or confidence) in the body of evidence for each outcome assessed. | 10-12, suppl. |
| **DISCUSSION** | | | |
| Discussion | 23a | Provide a general interpretation of the results in the context of other evidence. | 13-16 |
| | 23b | Discuss any limitations of the evidence included in the review. | 17 |
| | 23c | Discuss any limitations of the review processes used. | 17 |
| | 23d | Discuss implications of the results for practice, policy, and future research. | 13-16 |
| **OTHER INFORMATION** | | | |
| Registration and protocol | 24a | Provide registration information for the review, including register name and registration number, or state that the review was not registered. | 5 |
| | 24b | Indicate where the review protocol can be accessed, or state that a protocol was not prepared. | 5 |
| | 24c | Describe and explain any amendments to information provided at registration or in the protocol. | NA |
| Support | 25 | Describe sources of financial or non-financial support for the review, and the role of the funders or sponsors in the review. | 17 |
| Competing interests | 26 | Declare any competing interests of review authors. | 17 |
| Availability of data, code and other materials | 27 | Report which of the following are publicly available and where they can be found: template data collection forms; data extracted from included studies; data used for all analyses; analytic code; any other materials used in the review. | 17 |

**Supplementary Table S2. Search strategy for PubMed which was adapted for other databases.**

| | |
|---|---|
| **MEDLINE (via PubMed)** | ((((((((prone position[MeSH Terms]) OR ("prone positioning"[Title/Abstract])) OR ("Prone Positions"[Title/Abstract])) ) ) OR (PPV[Title/Abstract])) OR ("awake prone position"[Title/Abstract])) OR ("awake prone positioning"[Title/Abstract]))) AND ((((((((((("Oxygen inhalation therapy"[MeSH Terms]) OR ("Oxygen Inhalation"[Title/Abstract])) OR ("Oxygen therapy"[Title/Abstract])) OR ("Respiratory therapy"[Title/Abstract])) OR ("Non-intubated"[Title/Abstract])) OR ("Oxygen support"[Title/Abstract])) OR ("Oxygen supply"[Title/Abstract])) OR (awake[Title/Abstract])) OR ("spontaneous breath"[Title/Abstract])) OR (spontaneously breath[Title/Abstract])) OR ((((HFNC[Title/Abstract]) OR ("high-flow nasal cannula"[Title/Abstract])) OR ("high-flow nasal oxygen"[Title/Abstract])) OR ("high-flow oxygen"[Title/Abstract]))) OR ((((((Noninvasive Ventilation[MeSH Terms]) OR (NIV[Title/Abstract])) OR (NIPPV[Title/Abstract])) OR ("Non-Invasive Ventilation"[Title/Abstract])) OR ("Non invasive Ventilation"[MeSH Terms])) OR (NPPV[Title/Abstract]))) OR ((prone position[MeSH Terms]) OR ("prone positioning”[Title/Abstract]) OR ("Prone Positions”[Title/Abstract]) OR ("awake prone position”[Title/Abstract]) OR ("awake prone positioning”[Title/Abstract])) AND ((COVID19[MeSH Terms]) OR (SARS-CoV-2[MeSH Terms])) |

**Supplementary Table S3. Grading of recommendations assessment, development, and evaluation (GRADE) summary of findings.**

| Outcome | No. of participants (studies) | Effect estimate (95% CI) | Risk of bias | Inconsistency | Indirectness | Imprecision | Publication bias | Quality of Evidence (GRADE) |
|---|---|---|---|---|---|---|---|---|
| Need for intubation | 2385 (11) | RR 0.84 (0.74, 0.95) | Serious | Not serious | Not serious | Not serious | Not detected | ⊕⊕⊕⊖ Moderate |
| All-cause mortality | 2385 (11) | RR 0.93 (0.77, 1.11) | Serious | Not serious | Not serious | Serious | Not detected | ⊕⊕⊕⊖ Moderate |
| ICU length of stay | 980 (5) | MD 0.08 (-0.96, 1.12) | Not serious | Not serious | Not serious | Serious | N/A | ⊕⊕⊕⊖ Moderate |
| Hospital length of stay | 1677 (8) | MD 0.55 (-0.55, 1.66) | Serious | Not serious | Not serious | Not serious | N/A | ⊕⊕⊕⊖ Moderate |
| Need for escalating respiratory support | 1945 (9) | RR 0.97 (0.79, 1.20) | Serious | Not serious | Not serious | Not serious | N/A | ⊕⊕⊕⊖ Moderate |
| Ventilator-free days | 505 (3) | MD 3.36 (-7.20, 13.93) | Serious | Serious | Not Serious | Serious | N/A | ⊕⊖⊖⊖ Very low |

| | | | | | | | | |
|---|---|---|---|---|---|---|---|---|
| Adverse events | 2385 (11) | RR 1.29 (0.52, 3.21) | Serious | Serious | Not serious | Serious | Not detected | ⊕⊖⊖⊖<br><br>Very low |
| Serious adverse events | 1773 (3) | RR 1.60 (0.94, 2.73 | Not serious | Not serious | Not serious | Serious | N/A | ⊕⊕⊕⊖<br><br>Moderate |
| N/A = Not applicable | | | | | | | | |

**Supplementary Figure S1. Risk of bias assessment of included studies.**

| Study ID | D1 | D2 | D3 | D4 | D5 | Overall |
|---|---|---|---|---|---|---|
| Ehrmaan 2021 | + | + | + | + | + | + |
| Rosen 2021 | ! | + | + | + | + | ! |
| Taylor 2021 | ! | - | + | + | + | - |
| Johnson 2021 | + | ! | + | ! | + | ! |
| Fralick 2021 | ! | + | + | + | + | ! |
| Rampon 2022 | + | + | + | ! | + | ! |
| Harris 2021 | + | ! | - | ! | ! | ! |
| Kharat 2021 | ! | - | + | + | ! | - |
| Gad 2021 | ! | ! | - | + | ! | - |
| Alhazzani 2022 | + | + | + | + | + | + |
| Jayakumar 2021 | + | ! | + | ! | + | ! |

+ Low risk

! Some concerns

- High risk

D1 Randomisation process

D2 Deviations from the intended interventions

D3 Missing outcome data

D4 Measurement of the outcome

D5 Selection of the reported result

**Supplementary Figure S2. Funnel plot for risk of intubation.**

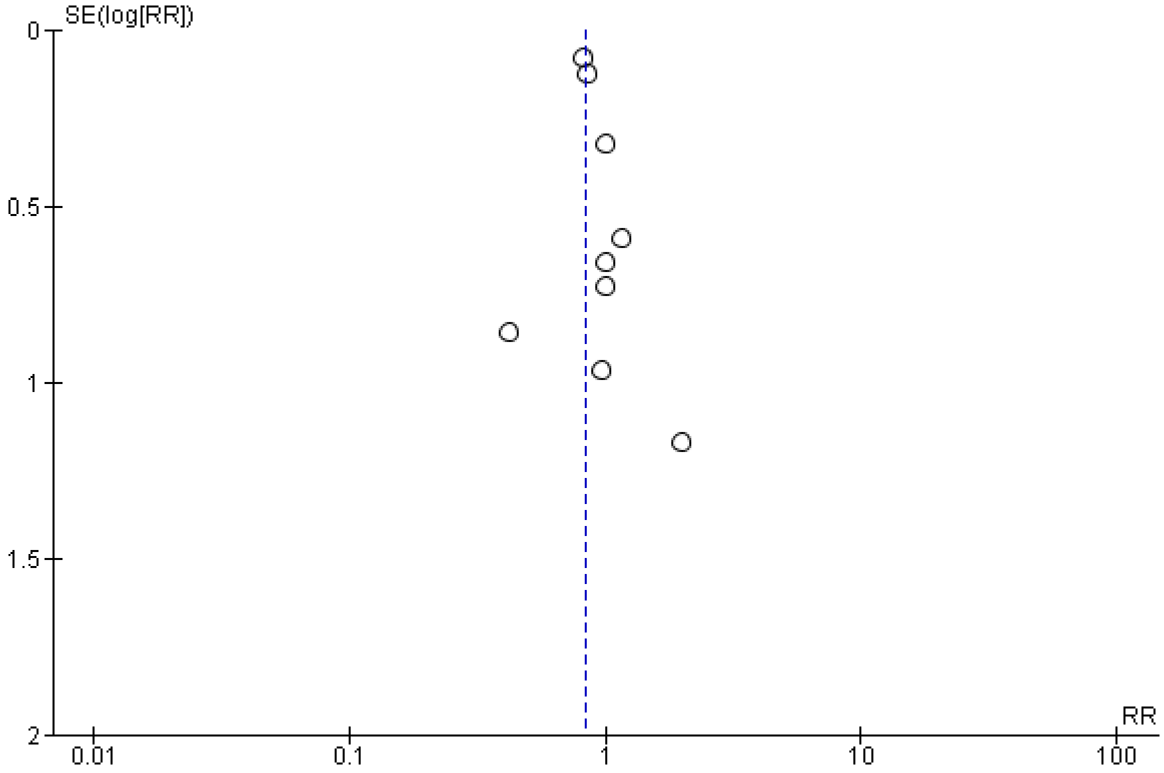

**Supplementary Figure S3. Subgroup analysis of risk of intubation for type of respiratory support before randomization.**

| Study or Subgroup | APP Events | APP Total | Control Events | Control Total | Weight | Risk Ratio M-H, Random, 95% CI |
|---|---|---|---|---|---|---|
| **2.3.1 Higher level of respiratory support** | | | | | | |
| Alhazzani, 2022 | 56 | 160 | 69 | 152 | 21.7% | 0.77 [0.59, 1.01] |
| Ehrmann, 2021 | 185 | 564 | 223 | 557 | 67.0% | 0.82 [0.70, 0.96] |
| Harris, 2021 | 1 | 5 | 1 | 8 | 0.3% | 1.60 [0.13, 20.22] |
| Rosén, 2021 | 12 | 36 | 13 | 39 | 4.0% | 1.00 [0.53, 1.90] |
| **Subtotal (95% CI)** | | **765** | | **756** | **92.9%** | **0.82 [0.71, 0.93]** |
| Total events | 254 | | 306 | | | |
| Heterogeneity: Tau² = 0.00; Chi² = 0.83, df = 3 (P = 0.84); I² = 0% | | | | | | |
| Test for overall effect: Z = 3.01 (P = 0.003) | | | | | | |
| **2.3.2 Conventional respiratory support** | | | | | | |
| Alhazzani, 2022 | 14 | 45 | 10 | 43 | 3.4% | 1.34 [0.67, 2.68] |
| Fralick, 2021 | 6 | 126 | 5 | 122 | 1.2% | 1.16 [0.36, 3.71] |
| Gad, 2021 | 3 | 15 | 3 | 15 | 0.8% | 1.00 [0.24, 4.18] |
| Harris, 2021 | 1 | 26 | 1 | 22 | 0.2% | 0.85 [0.06, 12.76] |
| Jayakumar, 2021 | 2 | 27 | 4 | 30 | 0.6% | 0.56 [0.11, 2.80] |
| Johnson, 2021 | 2 | 15 | 1 | 15 | 0.3% | 2.00 [0.20, 19.78] |
| Kharat, 2021 | 0 | 10 | 0 | 17 | | Not estimable |
| Rampon, 2022 | 2 | 159 | 4 | 134 | 0.6% | 0.42 [0.08, 2.26] |
| Taylor, 2021 | 0 | 27 | 0 | 13 | | Not estimable |
| **Subtotal (95% CI)** | | **450** | | **411** | **7.1%** | **1.07 [0.66, 1.73]** |
| Total events | 30 | | 28 | | | |
| Heterogeneity: Tau² = 0.00; Chi² = 2.57, df = 6 (P = 0.86); I² = 0% | | | | | | |
| Test for overall effect: Z = 0.27 (P = 0.78) | | | | | | |
| **Total (95% CI)** | | **1215** | | **1167** | **100.0%** | **0.83 [0.73, 0.95]** |
| Total events | 284 | | 334 | | | |
| Heterogeneity: Tau² = 0.00; Chi² = 4.52, df = 10 (P = 0.92); I² = 0% | | | | | | |
| Test for overall effect: Z = 2.83 (P = 0.005) | | | | | | |
| Test for subgroup differences: Chi² = 1.13, df = 1 (P = 0.29), I² = 11.9% | | | | | | |

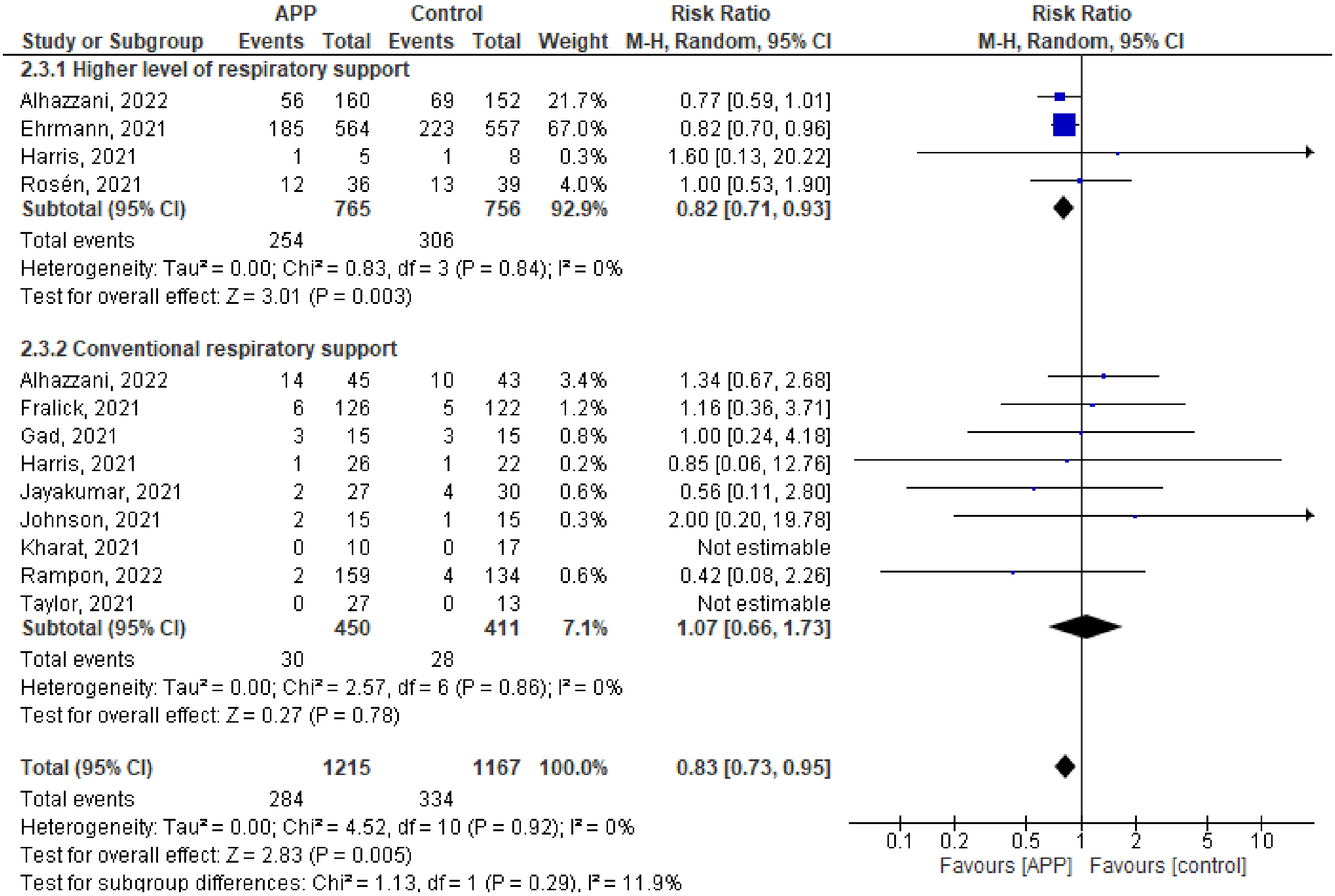

**Supplementary Figure S4. Subgroup analysis of risk of intubation for enrolment location.**

| | APP | | Control | | | Risk Ratio |
|---|---|---|---|---|---|---|
| **Study or Subgroup** | **Events** | **Total** | **Events** | **Total** | **Weight** | **M-H, Random, 95% CI** |
| **2.4.1 ICU** | | | | | | |
| Alhazzani, 2022 | 70 | 205 | 79 | 195 | 25.7% | 0.84 [0.65, 1.09] |
| Ehrmann, 2021 | 182 | 538 | 219 | 533 | 68.9% | 0.82 [0.70, 0.96] |
| Gad, 2021 | 3 | 15 | 3 | 15 | 0.8% | 1.00 [0.24, 4.18] |
| Jayakumar, 2021 | 4 | 30 | 4 | 30 | 1.0% | 1.00 [0.28, 3.63] |
| **Subtotal (95% CI)** | | **788** | | **773** | **96.5%** | **0.83 [0.73, 0.95]** |
| Total events | 259 | | 305 | | | |
| Heterogeneity: Tau² = 0.00; Chi² = 0.17, df = 3 (P = 0.98); I² = 0% | | | | | | |
| Test for overall effect: Z = 2.74 (P = 0.006) | | | | | | |
| **2.4.2 Non ICU** | | | | | | |
| Ehrmann, 2021 | 3 | 26 | 4 | 24 | 0.9% | 0.69 [0.17, 2.78] |
| Fralick, 2021 | 6 | 126 | 5 | 122 | 1.2% | 1.16 [0.36, 3.71] |
| Harris, 2021 | 2 | 31 | 2 | 30 | 0.5% | 0.97 [0.15, 6.44] |
| Johnson, 2021 | 2 | 15 | 1 | 15 | 0.3% | 2.00 [0.20, 19.78] |
| Kharat, 2021 | 0 | 10 | 0 | 17 | | Not estimable |
| Rampon, 2022 | 2 | 159 | 4 | 134 | 0.6% | 0.42 [0.08, 2.26] |
| Taylor, 2021 | 0 | 27 | 0 | 13 | | Not estimable |
| **Subtotal (95% CI)** | | **394** | | **355** | **3.5%** | **0.88 [0.44, 1.76]** |
| Total events | 15 | | 16 | | | |
| Heterogeneity: Tau² = 0.00; Chi² = 1.57, df = 4 (P = 0.81); I² = 0% | | | | | | |
| Test for overall effect: Z = 0.36 (P = 0.72) | | | | | | |
| **Total (95% CI)** | | **1182** | | **1128** | **100.0%** | **0.83 [0.73, 0.95]** |
| Total events | 274 | | 321 | | | |
| Heterogeneity: Tau² = 0.00; Chi² = 1.77, df = 8 (P = 0.99); I² = 0% | | | | | | |
| Test for overall effect: Z = 2.76 (P = 0.006) | | | | | | |
| Test for subgroup differences: Chi² = 0.03, df = 1 (P = 0.87), I² = 0% | | | | | | |

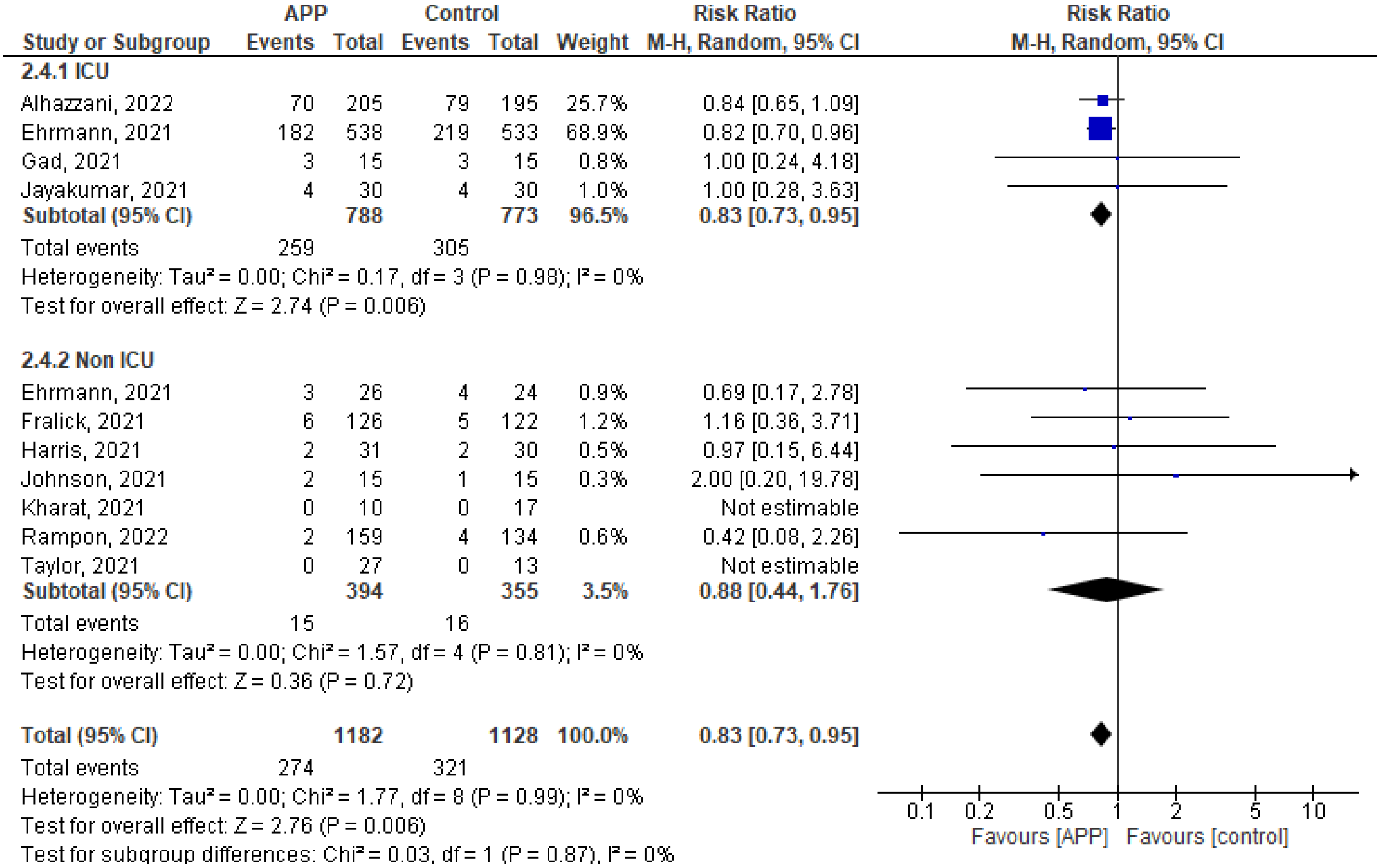

**Supplementary Figure S5. Meta-regression for risk of intubation by APP duration.**

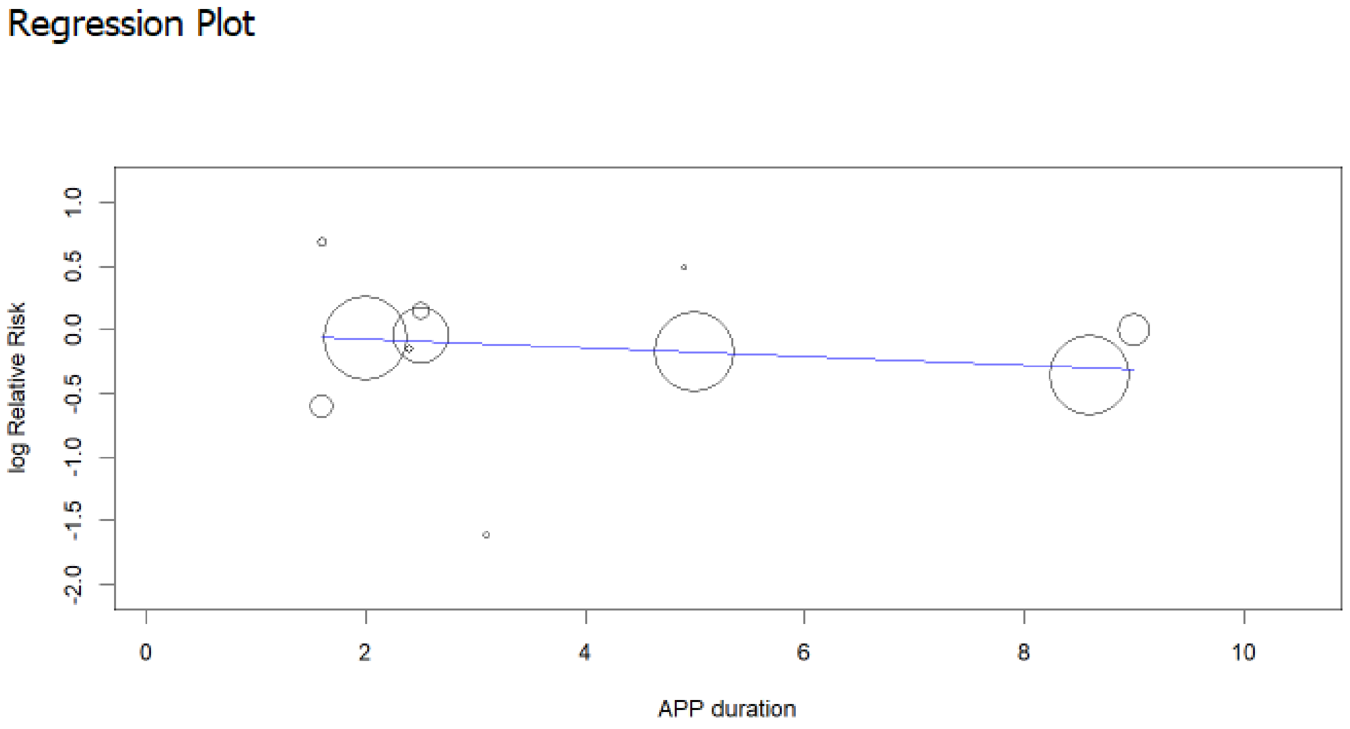

**Supplementary Figure S6. Funnel plot for all-cause mortality.**

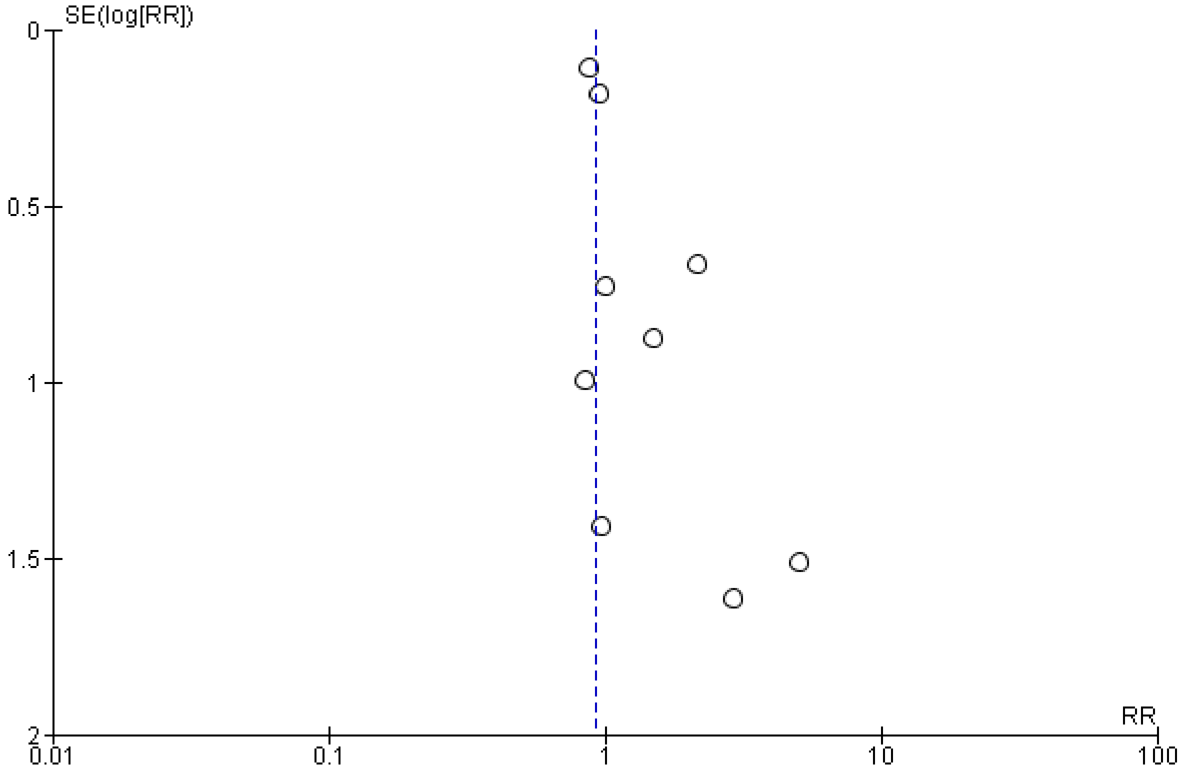

**Supplementary Figure S7. Subgroup analysis of all-cause mortality for type of respiratory support before randomization.**

| | APP | | Control | | | Risk Ratio |
|---|---|---|---|---|---|---|
| **Study or Subgroup** | **Events** | **Total** | **Events** | **Total** | **Weight** | **M-H, Random, 95% CI** |
| **2.1.1 Higher level of respiratory support** | | | | | | |
| Alhazzani, 2022 | 46 | 205 | 46 | 195 | 25.5% | 0.95 [0.66, 1.36] |
| Ehrmann, 2021 | 117 | 564 | 132 | 557 | 68.0% | 0.88 [0.70, 1.09] |
| Harris, 2021 | 1 | 5 | 0 | 8 | 0.4% | 4.50 [0.22, 93.07] |
| Rosén, 2021 | 6 | 36 | 3 | 39 | 1.9% | 2.17 [0.58, 8.03] |
| **Subtotal (95% CI)** | | **810** | | **799** | **95.8%** | **0.92 [0.76, 1.10]** |
| Total events | 170 | | 181 | | | |
| Heterogeneity: Tau² = 0.00; Chi² = 2.93, df = 3 (P = 0.40); I² = 0% | | | | | | |
| Test for overall effect: Z = 0.92 (P = 0.36) | | | | | | |
| **2.1.2 Conventional respiratory support** | | | | | | |
| Fralick, 2021 | 1 | 126 | 1 | 122 | 0.4% | 0.97 [0.06, 15.31] |
| Gad, 2021 | 3 | 15 | 3 | 15 | 1.6% | 1.00 [0.24, 4.18] |
| Harris, 2021 | 0 | 26 | 0 | 22 | | Not estimable |
| Jayakumar, 2021 | 2 | 27 | 2 | 30 | 0.9% | 1.11 [0.17, 7.35] |
| Johnson, 2021 | 2 | 15 | 0 | 15 | 0.4% | 5.00 [0.26, 96.13] |
| Kharat, 2021 | 0 | 10 | 0 | 17 | | Not estimable |
| Rampon, 2022 | 2 | 159 | 2 | 134 | 0.9% | 0.84 [0.12, 5.90] |
| Taylor, 2021 | 0 | 27 | 0 | 13 | | Not estimable |
| **Subtotal (95% CI)** | | **405** | | **368** | **4.2%** | **1.14 [0.47, 2.75]** |
| Total events | 10 | | 8 | | | |
| Heterogeneity: Tau² = 0.00; Chi² = 1.12, df = 4 (P = 0.89); I² = 0% | | | | | | |
| Test for overall effect: Z = 0.28 (P = 0.78) | | | | | | |
| **Total (95% CI)** | | **1215** | | **1167** | **100.0%** | **0.93 [0.77, 1.11]** |
| Total events | 180 | | 189 | | | |
| Heterogeneity: Tau² = 0.00; Chi² = 4.26, df = 8 (P = 0.83); I² = 0% | | | | | | |
| Test for overall effect: Z = 0.84 (P = 0.40) | | | | | | |
| Test for subgroup differences: Chi² = 0.22, df = 1 (P = 0.64), I² = 0% | | | | | | |

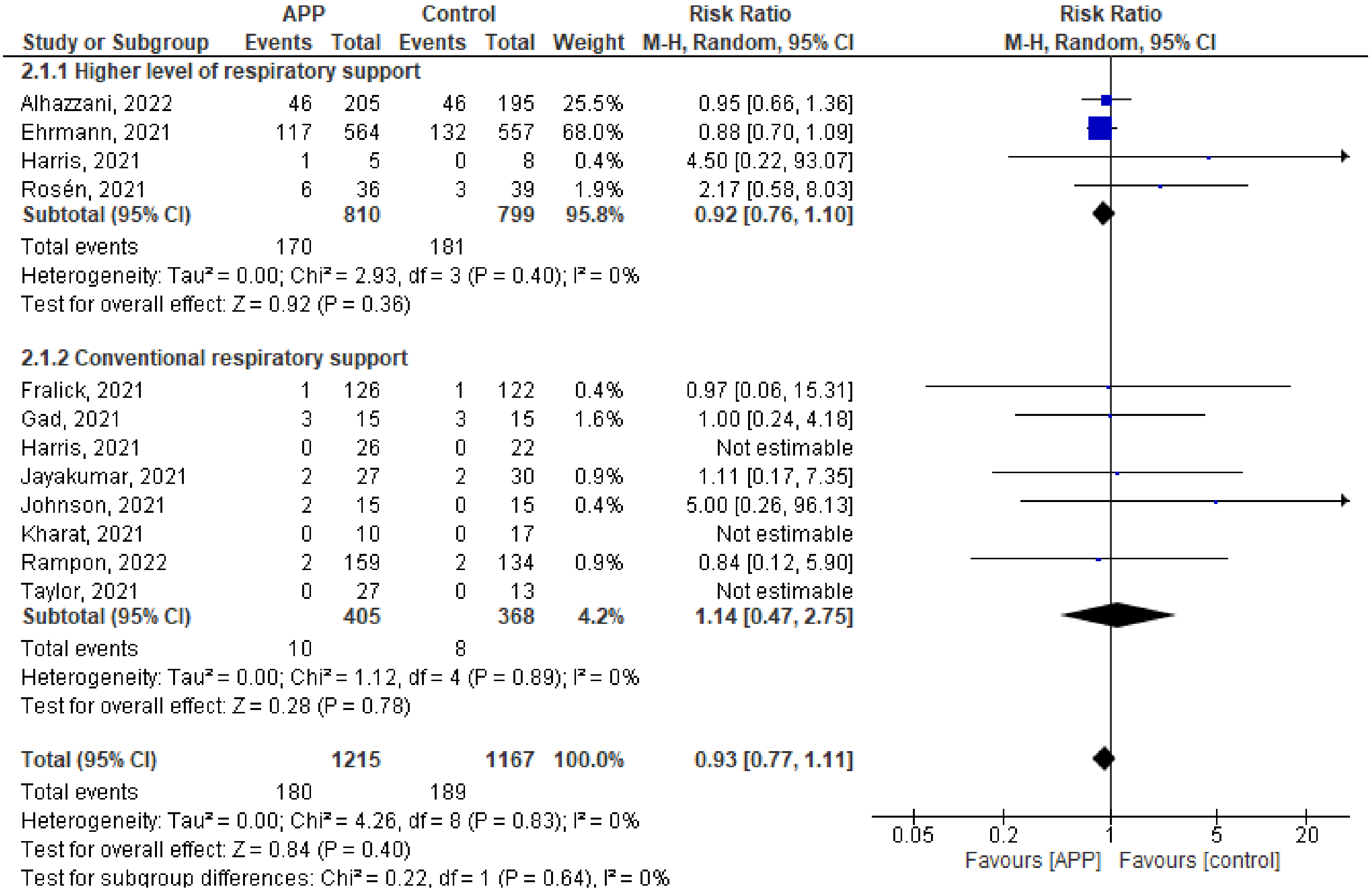

**Supplementary Figure S8. Subgroup analysis of all-cause mortality for enrolment location.**

| Study or Subgroup | APP Events | APP Total | Control Events | Control Total | Weight | Risk Ratio M-H, Random, 95% CI |
|---|---|---|---|---|---|---|
| **2.2.1 ICU** | | | | | | |
| Alhazzani, 2022 | 46 | 205 | 46 | 195 | 25.9% | 0.95 [0.66, 1.36] |
| Ehrmann, 2021 | 110 | 538 | 122 | 533 | 64.0% | 0.89 [0.71, 1.12] |
| Gad, 2021 | 3 | 15 | 3 | 15 | 1.6% | 1.00 [0.24, 4.18] |
| Jayakumar, 2021 | 3 | 50 | 2 | 30 | 1.1% | 0.90 [0.16, 5.08] |
| **Subtotal (95% CI)** | | **808** | | **773** | **92.6%** | **0.91 [0.75, 1.10]** |
| Total events | 162 | | 173 | | | |
| Heterogeneity: Tau² = 0.00; Chi² = 0.10, df = 3 (P = 0.99); I² = 0% | | | | | | |
| Test for overall effect: Z = 0.96 (P = 0.34) | | | | | | |
| **2.2.2 Non ICU** | | | | | | |
| Ehrmann, 2021 | 7 | 26 | 10 | 24 | 5.3% | 0.65 [0.29, 1.42] |
| Fralick, 2021 | 1 | 126 | 1 | 122 | 0.4% | 0.97 [0.06, 15.31] |
| Harris, 2021 | 1 | 31 | 0 | 30 | 0.3% | 2.91 [0.12, 68.66] |
| Johnson, 2021 | 2 | 15 | 0 | 15 | 0.4% | 5.00 [0.26, 96.13] |
| Kharat, 2021 | 0 | 10 | 0 | 17 | | Not estimable |
| Rampon, 2022 | 2 | 159 | 2 | 134 | 0.9% | 0.84 [0.12, 5.90] |
| Taylor, 2021 | 0 | 27 | 0 | 13 | | Not estimable |
| **Subtotal (95% CI)** | | **394** | | **355** | **7.4%** | **0.81 [0.41, 1.59]** |
| Total events | 13 | | 13 | | | |
| Heterogeneity: Tau² = 0.00; Chi² = 2.56, df = 4 (P = 0.63); I² = 0% | | | | | | |
| Test for overall effect: Z = 0.60 (P = 0.55) | | | | | | |
| **Total (95% CI)** | | **1202** | | **1128** | **100.0%** | **0.90 [0.75, 1.08]** |
| Total events | 175 | | 186 | | | |
| Heterogeneity: Tau² = 0.00; Chi² = 2.63, df = 8 (P = 0.96); I² = 0% | | | | | | |
| Test for overall effect: Z = 1.09 (P = 0.28) | | | | | | |
| Test for subgroup differences: Chi² = 0.10, df = 1 (P = 0.75), I² = 0% | | | | | | |

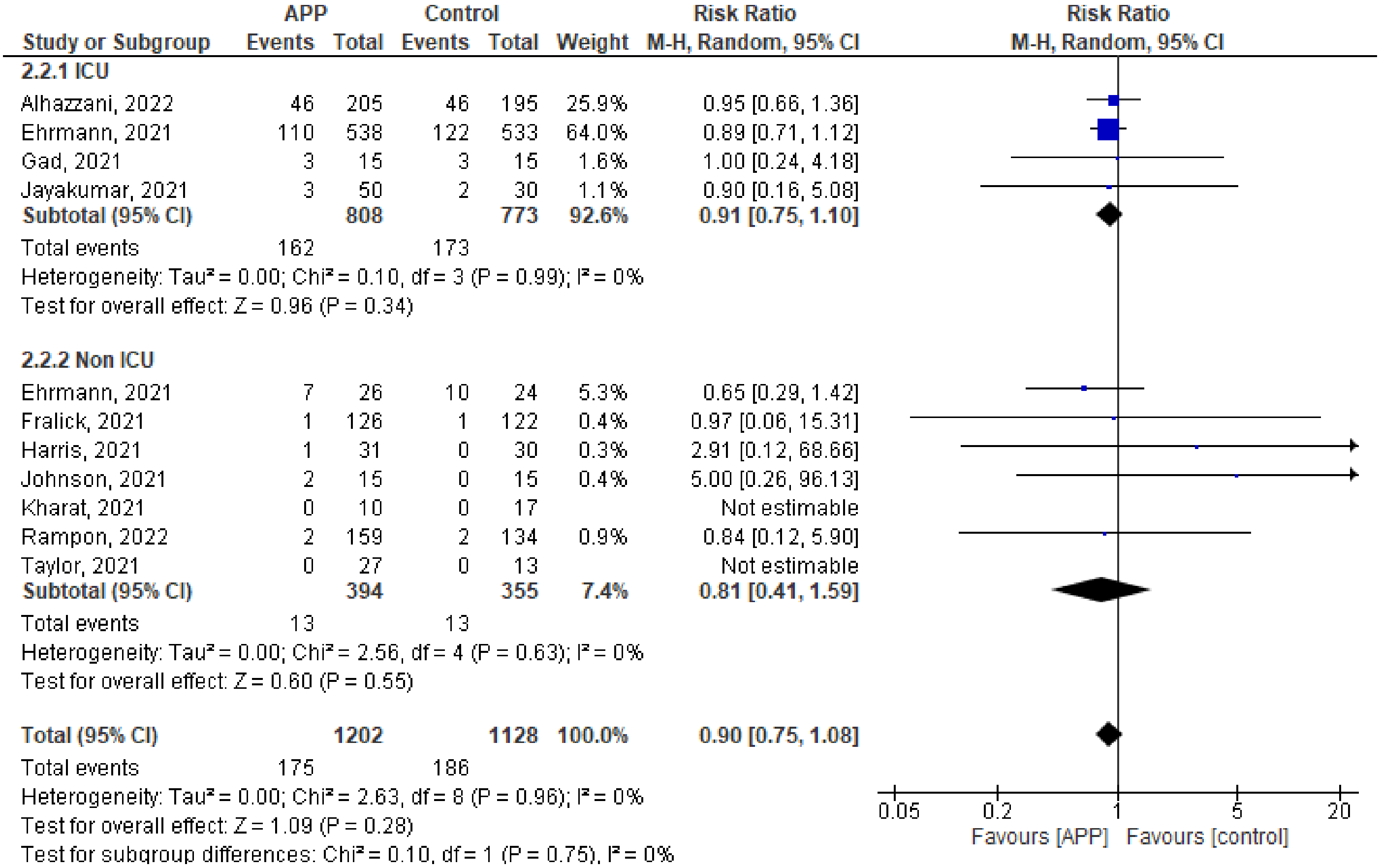

**Supplementary Figure S9. Meta-regression for risk of all-cause mortality by APP duration.**

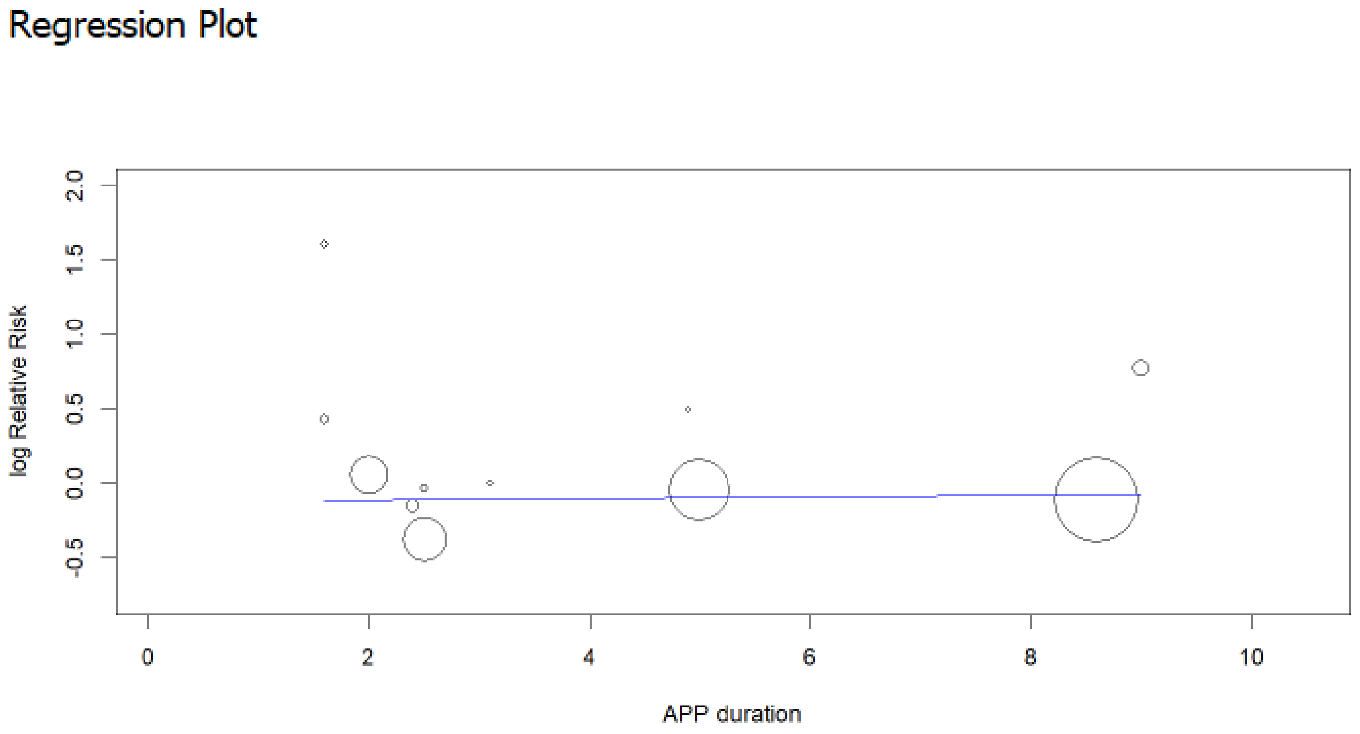

**Supplementary Figure S10. Effect of APP on length of ICU stay.**

| | APP | | | Control | | | | Mean Difference |
|---|---|---|---|---|---|---|---|---|
| Study or Subgroup | Mean | SD | Total | Mean | SD | Total | Weight | IV, Random, 95% CI |
| Ehrmann, 2021 | 11.71 | 11.07 | 360 | 11.92 | 9.55 | 394 | 40.6% | -0.21 [-1.69, 1.27] |
| Gad, 2021 | 8 | 3 | 15 | 7 | 2 | 15 | 28.5% | 1.00 [-0.82, 2.82] |
| Harris, 2021 | 2.6 | 4.9 | 31 | 3.6 | 4.2 | 30 | 19.0% | -1.00 [-3.29, 1.29] |
| Jayakumar, 2021 | 11.53 | 6.92 | 30 | 9.97 | 5.69 | 30 | 10.1% | 1.56 [-1.65, 4.77] |
| Rosén, 2021 | 11.13 | 17.12 | 36 | 16.03 | 17.5 | 39 | 1.8% | -4.90 [-12.74, 2.94] |
| **Total (95% CI)** | | | **472** | | | **508** | **100.0%** | **0.08 [-0.96, 1.12]** |

Heterogeneity: Tau² = 0.12; Chi² = 4.35, df = 4 (P = 0.36); I² = 8%
Test for overall effect: Z = 0.15 (P = 0.88)

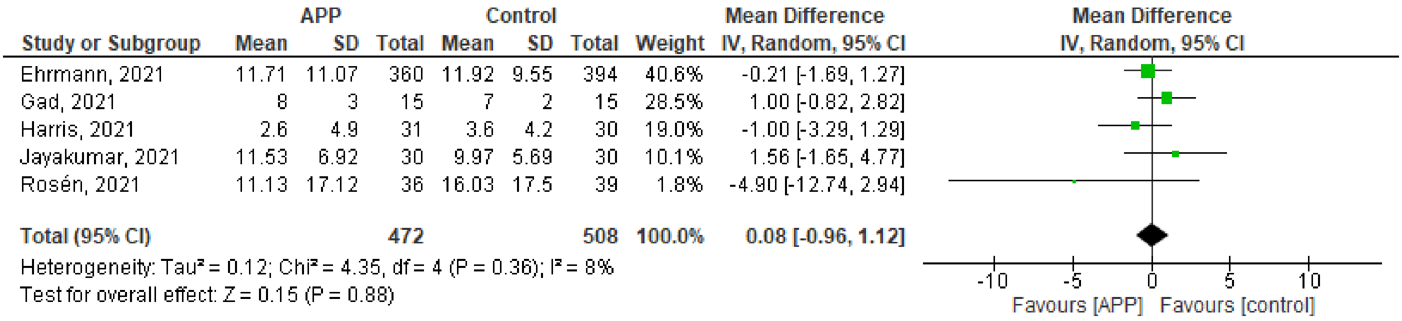

**Supplementary Figure S11. Effect of APP on length of hospital stay.**

| Study or Subgroup | APP Mean | APP SD | APP Total | Control Mean | Control SD | Control Total | Weight | Mean Difference IV, Random, 95% CI |
|---|---|---|---|---|---|---|---|---|
| Ehrmann, 2021 | 16.4 | 10.5 | 564 | 16.5 | 9.7 | 557 | 29.8% | -0.10 [-1.28, 1.08] |
| Gad, 2021 | 28 | 5 | 15 | 26 | 5 | 15 | 7.9% | 2.00 [-1.58, 5.58] |
| Harris, 2021 | 12.9 | 8.5 | 31 | 13.1 | 6.9 | 30 | 6.9% | -0.20 [-4.08, 3.68] |
| Johnson, 2021 | 6.58 | 6.3 | 15 | 4.24 | 1.33 | 15 | 9.2% | 2.34 [-0.92, 5.60] |
| Kharat, 2021 | 9.7 | 3.09 | 10 | 11 | 5.84 | 17 | 8.7% | -1.30 [-4.67, 2.07] |
| Rampon, 2022 | 4.84 | 5.36 | 159 | 3.67 | 3.71 | 134 | 32.3% | 1.17 [0.13, 2.21] |
| Rosén, 2021 | 17.29 | 8.51 | 36 | 23.94 | 20.74 | 39 | 2.3% | -6.65 [-13.73, 0.43] |
| Taylor, 2021 | 10.17 | 13.93 | 27 | 6.49 | 5.9 | 13 | 3.0% | 3.68 [-2.48, 9.84] |
| **Total (95% CI)** | | | **857** | | | **820** | **100.0%** | **0.55 [-0.55, 1.66]** |

Heterogeneity: $Tau^2 = 0.70$; $Chi^2 = 10.55$, df = 7 (P = 0.16); $I^2 = 34\%$
Test for overall effect: Z = 0.98 (P = 0.33)

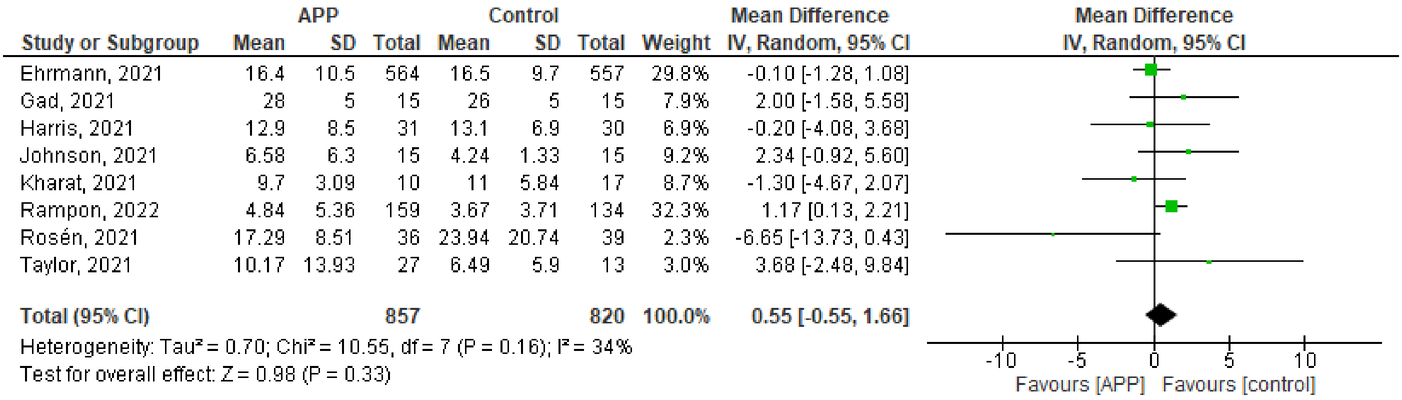

**Supplementary Figure S12. Effect of APP on need for escalating respiratory support.**

| | APP | | Control | | | Risk Ratio |
|---|---|---|---|---|---|---|
| Study or Subgroup | Events | Total | Events | Total | Weight | M-H, Random, 95% CI |
| Ehrmann, 2021 | 205 | 564 | 243 | 557 | 37.0% | 0.83 [0.72, 0.96] |
| Fralick, 2021 | 6 | 126 | 5 | 122 | 3.1% | 1.16 [0.36, 3.71] |
| Gad, 2021 | 3 | 15 | 3 | 15 | 2.1% | 1.00 [0.24, 4.18] |
| Harris, 2021 | 7 | 31 | 9 | 30 | 5.4% | 0.75 [0.32, 1.76] |
| Jayakumar, 2021 | 20 | 30 | 13 | 30 | 13.4% | 1.54 [0.95, 2.49] |
| Johnson, 2021 | 12 | 15 | 7 | 15 | 9.7% | 1.71 [0.94, 3.12] |
| Kharat, 2021 | 0 | 10 | 1 | 17 | 0.5% | 0.55 [0.02, 12.25] |
| Rampon, 2022 | 16 | 159 | 18 | 134 | 8.8% | 0.75 [0.40, 1.41] |
| Rosén, 2021 | 21 | 36 | 27 | 39 | 20.2% | 0.84 [0.60, 1.19] |
| **Total (95% CI)** | | **986** | | **959** | **100.0%** | **0.97 [0.79, 1.20]** |
| Total events | 290 | | 326 | | | |

Heterogeneity: $Tau^2 = 0.03$; $Chi^2 = 11.31$, df = 8 (P = 0.18); $I^2 = 29\%$
Test for overall effect: Z = 0.29 (P = 0.77)

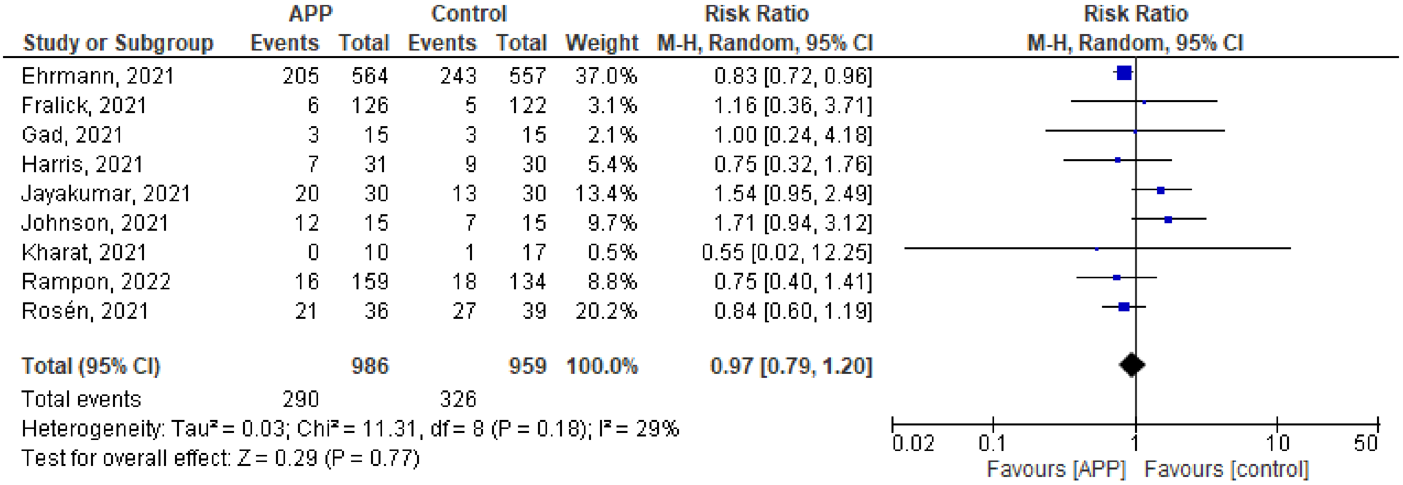

**Supplementary Figure S13. Effect of APP on ventilator-free days.**

| | APP | | | Control | | | | Mean Difference | Mean Difference |
|---|---|---|---|---|---|---|---|---|---|
| **Study or Subgroup** | **Mean** | **SD** | **Total** | **Mean** | **SD** | **Total** | **Weight** | **IV, Random, 95% CI** | **IV, Random, 95% CI** |
| Alhazzani, 2022 | 21.6 | 12.4 | 205 | 9.6 | 13.2 | 195 | 34.8% | 12.00 [9.49, 14.51] | |
| Johnson, 2021 | 24.27 | 8.07 | 15 | 27 | 3.26 | 15 | 33.5% | -2.73 [-7.13, 1.67] | |
| Rosén, 2021 | 24 | 13.3 | 36 | 23.7 | 14.07 | 39 | 31.7% | 0.30 [-5.89, 6.49] | |
| **Total (95% CI)** | | | **256** | | | **249** | **100.0%** | **3.36 [-7.20, 13.92]** | |

Heterogeneity: Tau² = 81.62; Chi² = 38.23, df = 2 (P < 0.00001); I² = 95%
Test for overall effect: Z = 0.62 (P = 0.53)

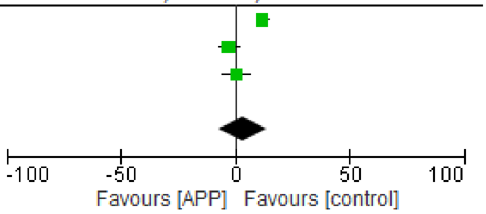

**Supplementary Figure S14. Effect of APP on the incidence of adverse events.**

| Study or Subgroup | APP Events | APP Total | Control Events | Control Total | Weight | Risk Ratio M-H, Random, 95% CI |
|---|---|---|---|---|---|---|
| Alhazzani, 2022 | 21 | 205 | 0 | 195 | 7.7% | 40.91 [2.50, 670.79] |
| Ehrmann, 2021 | 52 | 564 | 46 | 557 | 26.0% | 1.12 [0.76, 1.63] |
| Fralick, 2021 | 0 | 126 | 0 | 122 | | Not estimable |
| Gad, 2021 | 0 | 15 | 0 | 15 | | Not estimable |
| Harris, 2021 | 0 | 31 | 1 | 30 | 6.4% | 0.32 [0.01, 7.63] |
| Jayakumar, 2021 | 2 | 30 | 0 | 30 | 6.9% | 5.00 [0.25, 99.95] |
| Johnson, 2021 | 0 | 15 | 0 | 15 | | Not estimable |
| Kharat, 2021 | 6 | 10 | 0 | 17 | 7.8% | 21.27 [1.32, 341.84] |
| Rampon, 2022 | 14 | 159 | 28 | 134 | 24.4% | 0.42 [0.23, 0.77] |
| Rosén, 2021 | 5 | 36 | 10 | 39 | 20.8% | 0.54 [0.20, 1.43] |
| Taylor, 2021 | 0 | 27 | 0 | 13 | | Not estimable |
| **Total (95% CI)** | | **1218** | | **1167** | **100.0%** | **1.29 [0.52, 3.21]** |
| Total events | 100 | | 85 | | | |

Heterogeneity: Tau² = 0.80; Chi² = 24.98, df = 6 (P = 0.0003); I² = 76%

Test for overall effect: Z = 0.54 (P = 0.59)

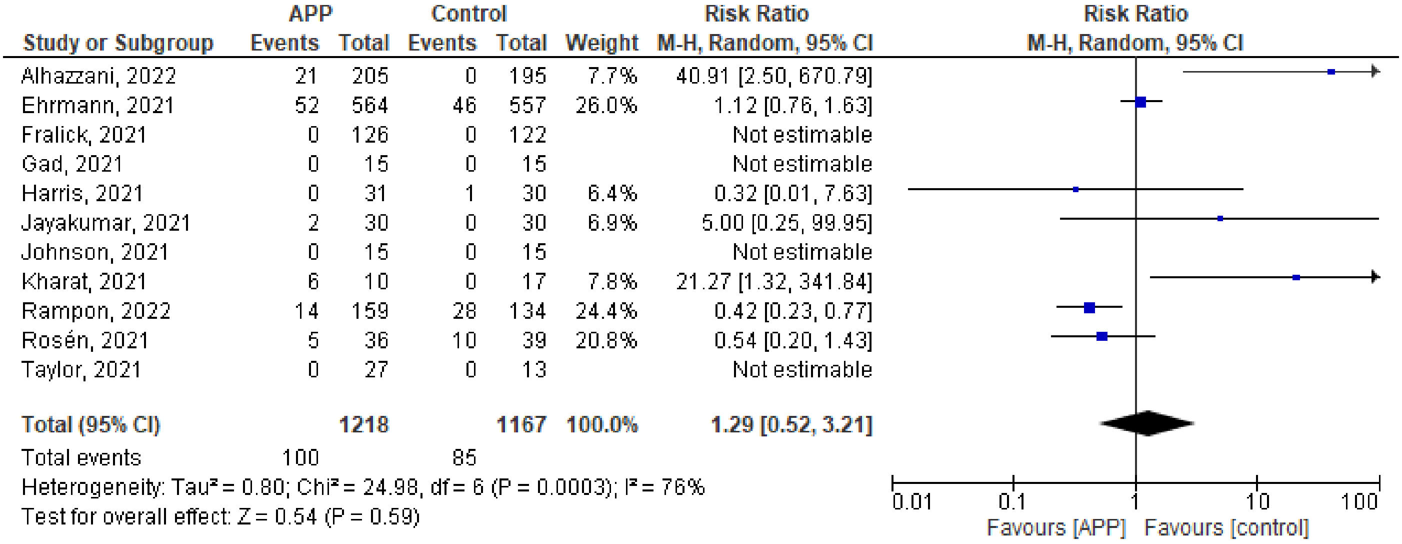

**Supplementary Figure S15. Effect of APP on the incidence of serious adverse events.**

| | APP | | Control | | | Risk Ratio |
|---|---|---|---|---|---|---|
| Study or Subgroup | Events | Total | Events | Total | Weight | M-H, Random, 95% CI |
| Alhazzani, 2022 | 0 | 205 | 0 | 195 | | Not estimable |
| Ehrmann, 2021 | 29 | 564 | 18 | 557 | 85.7% | 1.59 [0.89, 2.83] |
| Fralick, 2021 | 5 | 126 | 3 | 126 | 14.3% | 1.67 [0.41, 6.83] |
| **Total (95% CI)** | | **895** | | **878** | **100.0%** | **1.60 [0.94, 2.73]** |
| Total events | 34 | | 21 | | | |

Heterogeneity: Tau² = 0.00; Chi² = 0.00, df = 1 (P = 0.95); I² = 0%
Test for overall effect: Z = 1.73 (P = 0.08)

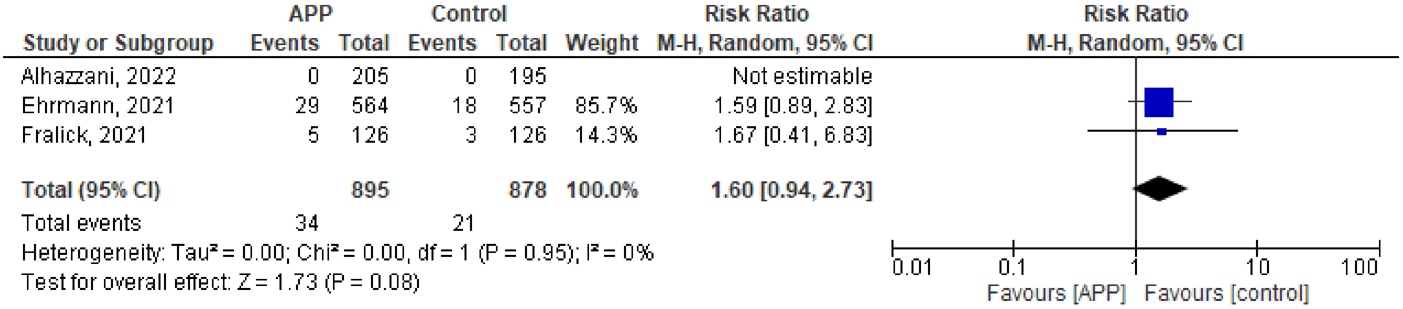

**Supplementary Figure S16. Funnel plot for the incidence of adverse events.**

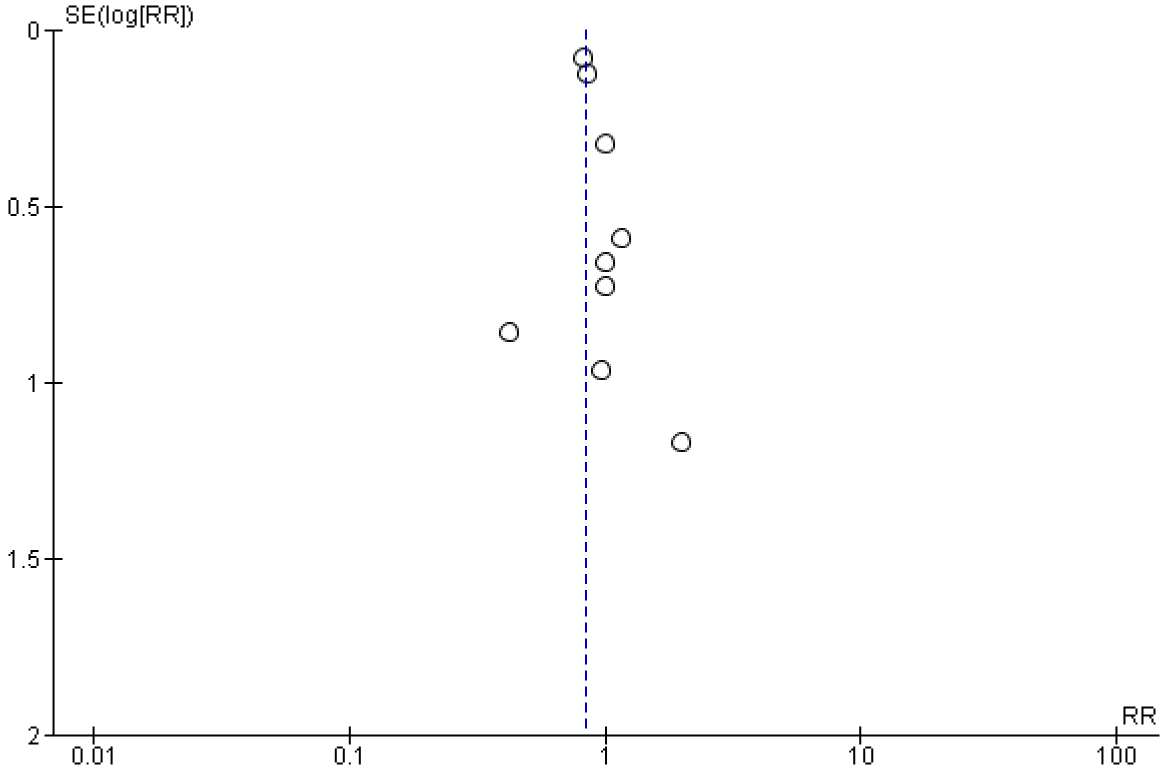

Supplement: Supplementary file 1 [file jcm-12-00926-s001.zip › jcm-2108768-supplementary.pdf]
